# Can Evidence from Genome-Wide Association Studies and Positive Natural Selection Surveys Be Used to Evaluate the Thrifty Gene Hypothesis in East Asians?

**DOI:** 10.1371/journal.pone.0110974

**Published:** 2014-10-22

**Authors:** Xuan-Han Koh, Xuanyao Liu, Yik-Ying Teo

**Affiliations:** 1 Department of Biological Sciences, National University of Singapore, Singapore, Singapore; 2 NUS Graduate School for Integrative Science and Engineering, National University of Singapore, Singapore, Singapore; 3 Saw Swee Hock School of Public Health, National University of Singapore, Singapore, Singapore; 4 Life Sciences Institute, National University of Singapore, Singapore, Singapore; 5 Department of Statistics and Applied Probability, National University of Singapore, Singapore, Singapore; 6 Genome Institute of Singapore, Agency for Science, Technology and Research, Singapore, Singapore; Shenzhen Institutes of Advanced Technology, China

## Abstract

Body fat deposition and distribution differ between East Asians and Europeans, and for the same level of obesity, East Asians are at higher risks of Type 2 diabetes (T2D) and other metabolic disorders. This observation has prompted the reclassifications of body mass index thresholds for the definitions of “overweight” and “obese” in East Asians. However, the question remains over what evolutionary mechanisms have driven the differences in adiposity morphology between two population groups that shared a common ancestor less than 80,000 years ago. The Thrifty Gene hypothesis has been suggested as a possible explanation, where genetic factors that allowed for efficient food-energy conversion and storage are evolutionarily favoured by conferring increased chances of survival and fertility. Here, we leveraged on the existing findings from genome-wide association studies and large-scale surveys of positive natural selection to evaluate whether there is currently any evidence to support the Thrifty Gene hypothesis. We first assess whether the existing genetic associations with obesity and T2D are located in genomic regions that are reported to be under positive selection, and if so, whether the risk alleles sit on the extended haplotype forms. In addition, we interrogate whether these risk alleles are the derived forms that differ from the ancestral alleles, and whether there is significant evidence of population differentiation at these SNPs between East Asian and European populations. Our systematic survey did not yield conclusive evidence to support the Thrifty Gene hypothesis as a possible explanation for the differences observed between East Asians and Europeans.

## Introduction

The past decade has seen a precipitous surge in obesity and diabetes globally, where the bulk of the increase has been in Asian populations such as China and India [1]. For example, a systematic review of 22 studies reported an increase in prevalence of Type 2 diabetes (T2D) in adults in China from 2.6% in 2000 to 9.7% in 2010 [2]. Epidemiologically, for the same body mass index (BMI), an East Asian individual is more susceptible to insulin resistance, and is more predisposed to body fat and visceral adiposity than a European individual [3]. The observations that East Asians tend to develop T2D at a less severe stage of obesity compared to Europeans have contributed in part to the reclassification of the definitions of “overweight” and “obese” for East Asians. Overweight and obese are defined as possessing a BMI ≥23 kg/m^2^ and BMI ≥28 kg/m^2^ respectively for East Asians, compared to corresponding thresholds of 25 kg/m^2^ and 30 kg/m^2^ for non-East Asians [3].

While the global surge in obesity and T2D has undoubtedly been attributed to the shift towards a high-fat high-sugar diet coupled with increasingly sedentary lifestyles, a fundamental question exists around the physiological differences in T2D etiology between East Asians and other populations. What are the molecular mechanisms that have resulted in the different types and location of adiposity, and what evolutionary processes could have driven these changes?

The Thrifty Gene hypothesis has been posited as a possible explanation for the steep increase in the prevalence of obesity and T2D in modern populations, particularly amongst East Asians. This hypothesizes that periods of famine in the history of modern humans presented a significant evolutionary force in favour of genes involved in fat storage, by increasing fertility and survival [4]. Even during fluctuating periods of abundance and scarcity, these individuals with metabolically thrifty genes were able to utilize food, store fat and gain weight more efficiently such that they were likely to survive better than those without the thrifty genes during periods of famine. The comparative perpetual abundance of food in modern societies means that the advantageous genes are preparing the hosts for famines that do not occur, which subsequently manifests as obesity and increases the risk to T2D [5]. A famine-like incident is believed to have occurred during the human migration to East Asia (and subsequently onwards to the Americas) in the last 20,000 years that is believed to have led to further enhancements of metabolically thrifty genes amongst East Asians than in the Europeans [6]–[9].

Presently, there have not been many concrete reports validating or refuting the Thrifty Gene hypothesis despite an ongoing debate and growing literature that focused on the role of natural selection in this hypothesis [5], [10]. One of the metrics to appraise the evidence of positive selection is by measuring the length of haplotypes in the genome. Recombination breaks down long haplotypes, and an uncharacteristically long haplotype at a locus often indicates that the particular genetic background has been preferred over other shorter haplotypes. This is thus indicative that a “hard sweep”, defined as a selection event with very high evolutionary fitness, has occurred.

The availability of high-density genetic data across multiple populations, coupled with recent discoveries from genome-wide association studies (GWAS) on obesity and T2D, confers an unprecedented opportunity to explore the Thrifty Gene hypothesis. Here, we combine findings from positive selection analyses and GWAS to evaluate whether there is any evidence to support the Thrifty Gene hypothesis in East Asians. This utilizes a recently developed method (haploPS) to locate genomic signatures of positive selection in humans [11], which additionally identifies the haplotype form that the advantageous allele sits on. This allows us to evaluate whether the selected haplotype form actually carries known variants that have been reported to increase the risk to obesity or T2D, which is vital given the reliance on tagging SNPs in GWAS to discover phenotypic associations.

Specifically, we adopted a four-step qualitative approach in evaluating the evidence to support the Thrifty Gene hypothesis in East Asians that is otherwise absent outside East Asia and the Americas. (i) We first examined the GWAS catalogue to identify SNPs that have been indisputably associated with obesity and T2D onset, and to compare these against the set of positive selection signals that were present only in the East Asian populations of the International HapMap Project [12] and the Singapore Genome Variation Project [13] (CHB, CHD, CHS, JPT). (ii) The selected haplotype forms were then checked to see whether they carried the reported risk alleles. (iii) In addition, we interrogated whether the reported risk alleles were the derived alleles by comparing against the chimpanzee genome sequence from dbSNP Build 138, as would have been supported under the Thrifty Gene hypothesis. (iv) Finally, we evaluated the degree of population differentiation using F_ST_ at the reported SNPs between East Asian and European populations, with the expectation of significant differentiation between the two ancestry groups if the East Asian-specific evolutionary events under the Thrifty Gene hypothesis did occur.

## Methods

### Datasets

We used the genome-wide genotype data for 11 populations from Phase 3 of the International HapMap Project (abbreviated subsequently as HapMap) [12], and for the three populations in the Singapore Genome Variation Project (SGVP) [13]. All 14 populations were genotyped on the Affymetrix SNP 6.0 and Illumina 1M genotyping microarrays. We only considered unrelated individuals from the HapMap and these consisted of: (i) 53 individuals of African ancestry in Southwest USA (ASW); (ii) 113 Utah residents with Northern and Western European ancestry from the CEPH collection (CEU); (iii) 84 Han Chinese from Beijing, China (CHB); (iv) 85 Chinese in Metropolitan Denver, Colorado (CHD); (v) 88 Gujarati Indians in Houston, Texas (GIH); (vi) 86 Japanese from Tokyo, Japan (JPT); (vii) 90 Luhyas from Webuye, Kenya (LWK); (viii) 50 individuals with Mexican ancestry in Los Angeles, California (MXL); (ix) 143 Maasai in Kinyawa, Kenya (MKK); (x) 88 Toscani in Italia (TSI); and (xi) 113 Yoruban from Ibadan, Nigeria (YRI). We considered a consensus set of 1,525,445 SNPs that was present across all eleven populations, and the phased haplotypes downloaded from the HapMap resource were thinned to consist of only this set of SNPs. The SGVP resource comprised 268 individuals from the three major ethnic groups in Singapore consisting of 96 Southern Han Chinese (CHS), 89 Southeast Asian Malays (MAS) and 83 South Asian Indians (INS). We considered the phased haplotypes of the post-QC set of autosomal SNPs that were available from the SGVP resource, which consisted of 1,584,040 SNPs for CHS, 1,580,905 SNPs for MAS and 1,583,454 SNPs for INS.

### Analysis of positive selection

Genomic evidence of positive selection was quantified with the use of the haploPS algorithm [11]. Briefly, haploPS explicitly locates uncharacteristically long haplotypes in the genome at a given frequency, which is defined against the distribution of haplotypes for that particular frequency across the genome by performing an exhaustive search across the SNPs. The length of each haplotype is measured by the genetic distance (in cM) spanned and by the number of SNPs on the haplotype. These two metrics are ranked against all other haplotypes across the genome of the same frequency in other to derive two empirical p-values: (i) the proportion of haplotypes that span a genetic distance at least as large as the target haplotype; and (ii) the proportion of haplotypes that span as many SNPs as the target haplotype. The haploPS score is defined as the product of these two empirical p-values and the total number of haplotypes across the genome at that particular frequency, and haplotypes with scores <0.05 are interpreted as exhibiting evidence of positive selection. This analysis is performed for each of the 14 populations, with the haplotype frequencies ranging from 0.05 to 0.95 in increments of 0.05. Since a site that is found to possess an uncharacteristically long haplotype at a particular frequency is similarly likely to possess long haplotypes at lower frequencies as well, we report only the evidence found at the highest haplotype frequency, and identify the specific haplotype form at this frequency as the one that is expected to carry the advantageous functional allele. Population-average recombination rates from the HapMap were used in the calculation of genetic distance [14].

### Quantifying similarity of positively selected haplotypes

A positive selection signal in a particular genomic region can be present across multiple populations, either by stemming from the same mutation event in the common ancestor of these multiple populations, or through convergent evolution where different mutation events in response to the same environmental trigger have happened independently in these populations. HaploPS quantifies the presence of positive selection by locating the longest haplotype form in a particular genomic region, and we can differentiate between the two scenarios above by comparing the selected haplotype forms from the multiple populations: in the former scenario where the selection seen in multiple populations is attributed to the same mutation event, we expect a significant degree of similarity between the haplotype forms; whereas in the latter scenario, we expect the advantageous alleles (either at the same site or at different sites) to have arisen on different haplotype backgrounds such that the longest haplotypes from different populations will exhibit a significant degree of discordance. As such, the haplotype similarity index (HSI), as described in the formulation of haploPS [11], is used to distinguish between the two scenarios, where a HSI >0.98 is indicative of a single mutation event in the common ancestor of the multiple populations, and a HSI <0.9 is indicative that convergent evolution is likely to have happened.

### Mapping GWAS signals to positively selected haplotypes

We considered the GWAS findings for obesity and T2D from the GWAS catalogue maintained by the National Human Genome Research Institute (NHGRI) at http://www.genome.gov/gwastudies/, which was accessed on 30^th^ June 2013. Specifically, we considered 56 loci for T2D from 31 GWAS studies and nine loci for obesity from four GWAS studies, where for each locus there was at least one SNP reported to be associated with the phenotype where the statistical significance of the association was more significant than 5×10^−8^ (**Tables S1, S2 in File S1**). Note that we did not require these associations to be specific to or have been reported in East Asian populations. In searching for uncharacteristically long haplotypes, haploPS explicitly identifies the specific haplotype form that is expected to be carrying the functional allele that confers an evolutionary advantage. We thus interrogated whether there was any evidence of positive selection in East Asian populations that overlapped with any of the 35 loci reported to be associated with T2D and obesity, and if so, whether the reported risk alleles were found on the selected haplotype forms in the East Asian populations. The loci are considered to be overlapping with the selection signals if they are located within the selection regions.

### Measurement of population differentiation

We used the locus-specific fixation index F_ST_ to quantify the extent of genetic differences between populations at a particular SNP [15]. This measures the divergence in the population-specific allele frequencies from the population-averaged allele frequency, and is defined as the ratio of the observed variance for the allele frequencies across the populations to the maximum possible variance under the global allele frequency.

## Results

Starting with the list of 405 positively selected genomic regions in the 14 populations from HapMap and SGVP, we identified six T2D-associated index SNPs in or around nine genes from the GWAS catalogue (**Table 1**) that overlapped with evidence of positive selection in the East Asian populations in HapMap and SGVP (**Table 2**) [16]–[24]. Three of these SNPs were located in six genes (*ARF5, PAX4, SND1, GCC1, C2CD4A, C2CD4B*) that were identified to be associated with T2D in only East Asian populations, while *HHEX* was common to both East Asian and Europeans, and two genes (*THADA*, *IDE*) were specific to the Europeans. In particular, we noted that the specific allele for rs7578597 in *THADA* that was identified to increase the risk of T2D was almost fixed in the two East Asian populations (CHD, CHS). Strikingly, even though we started with the intention to consider both obesity and T2D-associated loci, only the T2D-associated loci met our criteria for being East Asian specific in both trait association and positive selection signals.

**Table 1:** Positively selected Type 2 diabetes associated SNPs from genome-wide association studies (GWAS).

SNP	Chr	Pos. NCBI36.3 (bp)	No-riskallele	Riskallele	Risk allelefrequency	Nearestgene(s)	*p*-value	Oddsratio	Reported inEast Asians	Reported inEuropeans
rs7578597	2	43,586,327	C	T^a^	0.9	THADA	1×10^−9^	1.15	N	Y
rs10229583	7	127,034,139	A	G^a^	0.825	ARF5, PAX4, SND1	2×10^−10^	1.14	Y	N
rs6467136	7	126,952,194	A	G^a^	0.79	GCC1, PAX4	5×10^−11^	1.11	Y	N
rs5015480	10	94,455,539	T	C^a^	NR	HHEX, IDE	1×10^−15^	1.18	N	Y
rs1111875	10	94,452,862	T^a^	C	0.52	HHEX	7×10^−12^	1.21	Y	Y
rs7172432	15	60,183,681	G^a^	A	0.58	C2CD4A, C2CD4B	9×10^−14^	1.11	Y	N

Summary of six SNPs reported to be associated with Type 2 diabetes from the GWAS catalogue maintained by the National Human Genome Research Institute (NHGRI), accessed on 30^th^ June 2013, that are located in genomic regions that are putatively under positive selection in at least one of 14 populations from the International HapMap Project and the Singapore Genome Variation Project.

a Ancestral allele.

**Table 2:** Long haplotype regions (specific to East Asians) containing trait associated SNPs.

Chr	Start position	End position	HaploPS score	Pop.	Pos. NCBI 36.3 (bp)	rsID	Risk allele	Hap. freq.	All. freq.	Found on hap.	Nearest gene(s)
2	43,282,562	43,910,360	0.017	CHD	43,586,327	rs7578597 (3′ UTR)	T^a^	0.45	0.994	Y	THADA
2	43,301,270	43,958,429	0.008	CHS	43,586,327	rs7578597 (3′ UTR)	T^a^	0.45	1	Y	THADA
7	126,647,066	127,610,096	0.021	CHB	127,034,139	rs10229583 (downstream)	G^a^	0.40	0.798	Y	ARF5, PAX4, SND1
7	126,647,066	127,610,096	0.021	CHB	126,952,194	rs6467136 (intergenic)	G^a^	0.40	0.775	Y	GCC1, PAX4
7	126,716,840	127,589,178	0.022	CHS	127,034,139	rs10229583 (downstream)	G^a^	0.45	0.775	Y	ARF5, PAX4, SND1
7	126,716,840	127,589,178	0.022	CHS	126,952,194	rs6467136 (intergenic)	G^a^	0.45	0.801	Y	GCC1, PAX4
7	126,720,180	127,631,331	0.024	CHD	127,034,139	rs10229583 (downstream)	G^a^	0.45	0.829	Y	ARF5, PAX4, SND1
7	126,720,180	127,631,331	0.024	CHD	126,952,194	rs6467136 (intergenic)	G^a^	0.45	0.780	Y	GCC1, PAX4
7	126,735,606	127,628,256	0.015	JPT	127,034,139	rs10229583 (downstream)	G^a^	0.50	0.866	Y	ARF5, PAX4, SND1
7	126,735,606	127,628,256	0.015	JPT	126,952,194	rs6467136 (intergenic)	G^a^	0.50	0.818	Y	GCC1, PAX4
10	92,947,202	95,385,717	0.032	JPT	94,455,539	rs5015480 (downstream)	C^a^	0.05	0.186	N	HHEX, IDE
10	92,947,202	95,385,717	0.032	JPT	94,452,862	rs1111875 (intergenic)	C	0.05	0.349	N	HHEX
15	61,999,328	62,941,185	0.033	CHS	60,183,681	rs7172432 (intergenic)	A	0.60	0.330	N	C2CD4A, C2CD4B
15	62,122,278	62,938,688	0.003	JPT	60,183,681	rs7172432 (intergenic)	A	0.70	0.552	N	C2CD4A, C2CD4B
15	62,303,982	62,934,451	0.012	CHD	60,183,681	rs7172432 (intergenic)	A	0.80	0.631	N	C2CD4A, C2CD4B

Three SNPs from three genomic regions associated with T2D in or near *ARF5, PAX4, SND1, GCC1, C2CD4A,* and *C2CD4B* genes, which meet the criteria that both the evidence of T2D association and positive selection (haploPS score <0.05) were specific to only East Asians. The allele that increases the risk of T2D is identified and interrogated whether it (i) matches the ancestral allelic form on the chimpanzee genome, and/or (ii) sits on the positively selected haplotype form.

a Ancestral allele.

**Figure 1:**
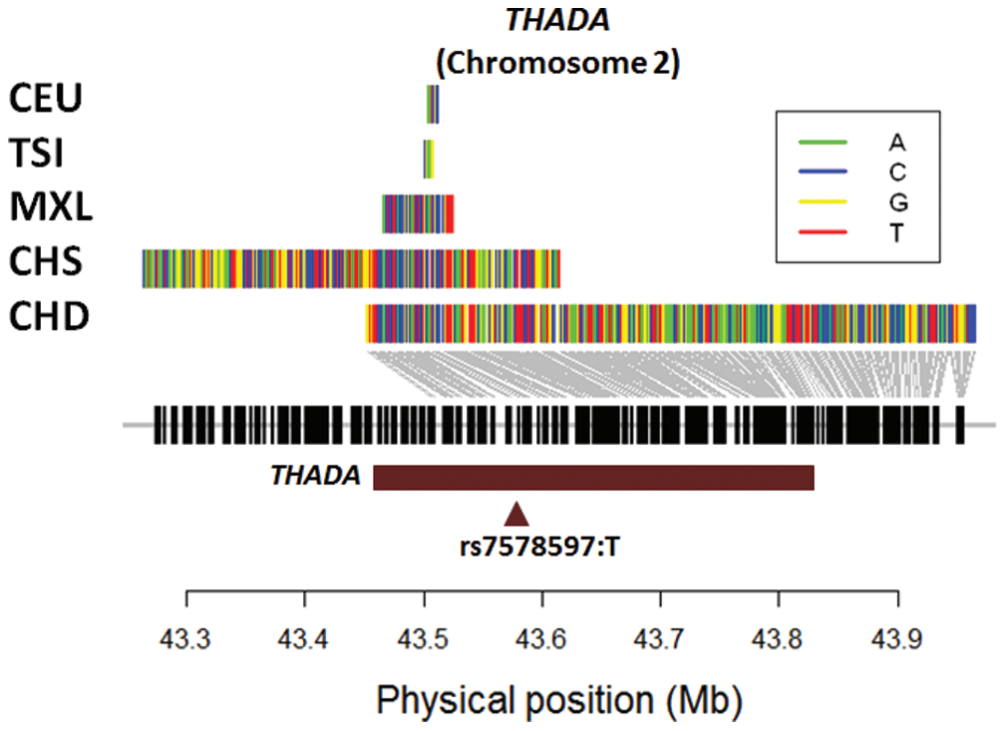
Long haplotypes around *THADA* in East Asian and European populations. Illustration of haplotype forms identified by haploPS that span the longest genetic distances around *THADA* (brown horizontal bar) on chromosome 2. Uncharacteristically long haplotypes were found at the same frequency of 45% in two East Asian populations (CHD, CHS) to span *THADA*, whereas at the same frequency, the longest haplotypes present in European populations (CEU, TSI, MXL) were comparatively much shorter. The haplotypes for the two East Asian populations also carried the thymine allele at rs7578597 that has been reported in association studies to increase the risk to Type 2 diabetes.

For *THADA*, the T2D risk allele was located on the positively selected haplotype identified by haploPS (**Figure 1**, **Table 2**). The evidence of positive selection was present in two of the four East Asian populations (CHD, CHS), and the selected haplotype forms from both populations were identical with a haplotype similarity index (HSI) of 1.00. We observed that haploPS inferred the frequency of the selected haplotype to be 45% for both populations, although the frequencies of the risk alleles were at 99.4% and 100% in CHD and CHS respectively. However, it was noted that the associated risk allele was in fact an ancestral allele identical to that found in the chimpanzee genome.

**Figure 2:**
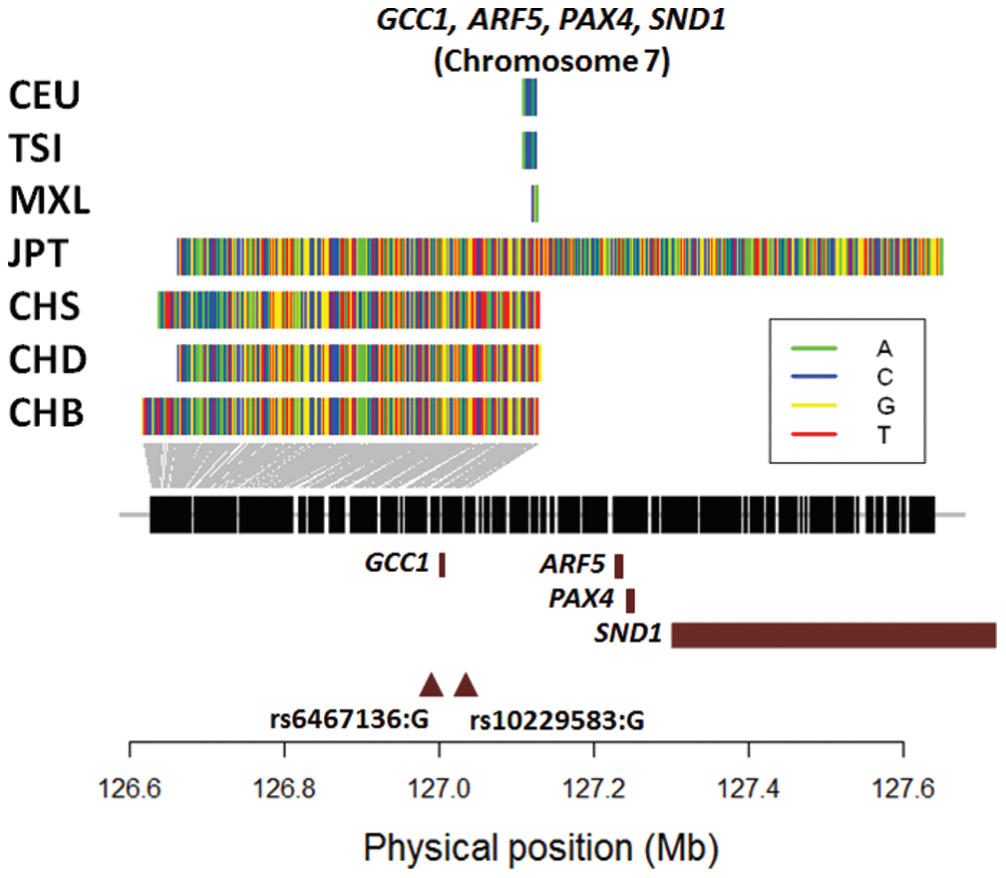
Long haplotypes around two T2D-associated SNPs in East Asian and European populations. Illustration of haplotype forms identified by haploPS that span the longest genetic distance around two SNPs rs6467136 and rs10229583 that are located around a cluster of four genes on chromosome 7 (brown horizontal bars: *GCC1*, *ARF5*, *PAX4*, *SND1*). The figure shows the uncharacteristically long haplotypes that were present at a haplotype frequency of 40% in the four East Asian populations (CHB, CHD, CHS, JPT), together with the longest haplotypes present in the European populations (CEU, TSI, MXL) that were comparatively much shorter and did not yield significant evidence of positive selection. The haplotypes for the four East Asian populations carried both risk alleles at the two SNPs that have been reported in association studies to increase the risk to Type 2 diabetes.

We observed the same evidence for the two index SNPs (rs10229583, rs6467136) located in the cluster of genes (*ARF5*, *PAX4*, *SND1*, *GCC1*) on chromosome 7, where the specific risk alleles were located on the positively selected haplotypes identified by haploPS (**Figure 2**, **Table 2**). The selection evidence was present in all four East Asian populations (CHB, CHD, CHS, JPT), and the selected haplotype forms from all four populations were identical with a HSI of 1.00. HaploPS inferred the frequency of the selection to be between 40% and 50% in the four populations, although the frequencies of the risk alleles ranged between 77% and 86%. Both risk alleles were ancestral and common with the chimpanzee genome.

The risk alleles for the remaining three index SNPs (rs5015480, rs1111875, rs7172432) did not sit on the positively selected haplotype. For rs7172432 on chromosome 15, it was also clear from the frequencies of the risk allele and the selected haplotypes that the selected haplotypes did not uniquely carry the risk or the protective allele (**Table 2**, **Figure S1 in File S1**).

With the evidence of positive selection and trait association converging for only *THADA* and the gene cluster on chromosome 7, the analysis of genetic differentiation thus focused on only the three relevant index SNPs. We observed that while both rs7578597 and rs10229583 were not significantly differentiated between East Asian and European populations, rs6467136 exhibited F_ST_ scores that were in excess of 8.2% (**Table 3**), indicating significant degree of genetic differentiation.

**Table 3:** F_ST_ scores between pairs of East Asian and European populations at three index SNPs around *THADA* and on chromosome 7 in the cluster of genes containing *GCC1*, *ARF5*, *PAX4* and *SND1*.

F_ST_ score	CHB-CEU	CHB-TSI	CHB-MXL	CHD-CEU	CHD-TSI	CHD-MXL	JPT-CEU	JPT-TSI	JPT-MXL
rs7578597	–	–	–	0.057	0.024	0.004	–	–	–
rs10229583	0.012	0.007	<0.001	0.011	0.006	<0.001	0.024	0.016	0.006
rs6467136	0.082	0.131	0.113	0.085	0.135	0.116	0.113	0.168	0.147

Population abbreviations represent: CEU – Utah residents with Northern and Western European ancestry from the CEPH collection; CHB – Han Chinese in Beijing, China; CHD – Chinese in Metropolitan Denver, Colorado; JPT – Japanese in Toyko, Japan; MXL – Mexican ancestry in Los Angeles, California; TSI – Toscani in Italia.

## Discussion

We started with the intention to evaluate whether genetics can explain the disparity in risk to T2D and other cardiovascular diseases between East Asians and Europeans, where for the same level of obesity as measured by BMI, East Asians are more likely than their European counterparts to experience a cardiac event or to be diabetic. One possible explanation centers around the Thrifty Gene hypothesis, and we attempted to evaluate whether current findings from GWAS and genetic surveys of positive selection can provide the genetic evidence to support this hypothesis. We observed that only six T2D-associated loci were found in regions undergoing positive selection in East Asian populations, and while the risk alleles of three of these loci sat on the positively selected haplotypes, they were all ancestral alleles that, despite acknowledging the reliance of GWAS on identifying tagging SNPs, did not conform to the notion that these alleles are derived from specific mutation events that have occurred recently to confer greater survivability. Similar to a previous report [25], we did not find consistent evidence to unambiguously support the Thrifty Gene hypothesis as it pertains to East Asians.

There has been considerable confusion as to what constitutes a ‘thrifty gene’. While it is most commonly associated with a metabolic trait related to the frugal utilization of fuel [7], various researchers have broadened the concept to encompass multiple notions of thrift, such as behavioral and physiological traits that might be adaptive during periods of food shortages [7], [26]. In this paper, we adopted the definition of ‘thrift’ as five broad categories that favor sustained positive energy balance as suggested by Bouchard [27], encompassing (a) metabolism or thermogenesis, (b) regulation of appetite, (c) physical inactivity, (d) lipid oxidization, and (e) lipid storage capacity of adipocytes, for practical purposes of testability. As such, the central assumption is that individuals possessing thrifty genes may exhibit high adiposity in modern society, and thus be identified among populations of people with obesity and/or type 2 diabetes. While obesity and type 2 diabetes are not perfect surrogate measures of the modern day effects of metabolic thrift, they represent objective measures of the biological system that can be tested and compared across populations.

It is interesting to note that, despite explicitly searching for obesity and T2D-associated loci, none of the obesity-associated SNPs displayed evidence of position selection that was specific to East Asians even though obesity is commonly regarded as upstream of T2D pathogenesis and would have been a better surrogate measure for the presence of thrifty genes. Although obesity is a major predictor of T2D, most obese individuals do not develop T2D, and conversely T2D patients are not necessarily obese. The complexity of the interaction between both diseases illustrates the difficulty in using T2D as a surrogate for the modern day effect of thrifty genes.

A gene that shows evidence of positive selection and an association with T2D is not necessarily a thrifty gene, as it may have been selected for reasons other than thrift. For example, the thymine allele of rs7903146 in *TCF7L2* increases T2D risk with an odds ratio of 1.37 [28], representing the T2D susceptibility gene with the largest effect size discovered to date that has been successfully replicated across multiple populations. However, the HapB_T2D_ haplotype containing the risk allele is found to be negatively associated with BMI in T2D patients and is not the variant that displays evidence of recent positive selection, contrary to predictions of the thrifty gene hypothesis [29]. If thrifty genes indeed exist, functional evidence that the T2D risk alleles are involved in a mechanism consistent with thrift is essential.

One challenge to testing the Thrifty Gene theory is the lack of clarity behind the mechanisms linking obesity, T2D, and insulin resistance. It has been proposed that the phenotypic expression of the thrifty gene may be hyperinsulinemia or insulin resistance [30], although this appears to be paradoxical as a decrease in insulin sensitivity suggests a negative impact on fat storage ability. For normal individuals without pancreatic islet β-cell dysfunction, the β-cells increase insulin secretion sufficiently to compensate for the reduced sensitivity to insulin action, thereby maintaining normal glucose tolerance [31]. It is hypothesized that this increase in blood insulin is responsible for a greater net tendency towards fat storage. However, some have proposed that the evidence behind insulin resistance as the phenotypic expression of the thrifty gene is inconsistent [32], citing evidence that Pima Indians have the greatest prevalence of diabetes in the world and ought to be an obvious candidate for carriers of thrifty genes given the population genetic history, except that in non-diabetic Pima Indians, insulin resistance is instead associated with a reduced risk of weight gain [33] which is contrary to what one would expect under the Thrifty Gene hypothesis.

One proposed mechanism linking obesity, insulin resistance, and T2D suggests that a defect in the ability of β-cells to secrete insulin is sufficient to lead to decreased insulin signaling in the hypothalamus, resulting in increased food intake and thus weight gain [34]. In this way, a thrifty allele that causes β-cell dysfunction and results in decreased insulin secretion can potentially be selected for thrift through its effects on increased food intake, consistent with the Thrifty Gene hypothesis. However, a decrease in insulin secretion also impairs the storage of glucose as triglycerides in adipocytes, leading to increased lipolysis and elevated plasma non-esterified fatty acids (NEFA) levels [31]. The increase in NEFA and glucose levels in the plasma, together with increased food intake due to brain signaling processes can result in insulin resistance, further aggravating β-cell function [31]. Interestingly, the convergence of events towards increased insulin resistance may hint at thrifty genes with different phenotypic expressions that function along a similar pathway. It thus remains an open question as to whether thrifty genes directly affect β-cell function, insulin resistance, fat storage upstream of any T2D-related event, or if a more plausible thrifty genotype exists where numerous genes involved in different functions at various points along the carbohydrate and fat regulation pathway account for an aggregate effect towards fat storage. Even then, a possibility maintains that thrift is also accounted for by mechanisms other than increased fat storage.

Though it remains to be demonstrated that the genes at which the index SNPs are located in are functionally responsible for the GWAS signals for obesity and T2D, there is a great amount of evidence supporting the roles of the identified genes, particularly for *THADA* and *PAX4*. The *THADA* risk allele is linked to decreased glucagon-like peptide-1-induced and arginine-induced insulin release [35], while studies have shown that the early expression of *PAX4* is involved in the differentiation of β-cells in the pancreas, thus affecting insulin secretion [36], [37]. It has to be noted that both genes affect pancreatic insulin secretion and/or plasma insulin levels that is upstream of fat storage. Ideally, we would expect a thrifty allele to be directly involved in efficient fat storage. However, it remains to be seen if genes that affect fat storage indirectly have less of an impact on fertility and/or survival, and thus selection.

Perhaps one of the most plausible explanations on the lack of evidence thus far for the Thrifty Gene hypothesis is the fact that the search for genomic signatures of positive selection with recent genome-wide data has primarily focused on locating hard sweeps, given the reliance on methods that capitalise on variance in allele frequencies or the presence of long haplotypes. These methods are capable of identifying selection signals that leave distinctive imprints in the genome, but are unfortunately poorly powered to locate evidence arising from polygenic soft sweeps. This is compounded by the fact that obesity and T2D are complex phenotypes, where even the genetic determinants are multi-factorial and exert at best a modest impact, unlike the classical situation where genetic mutations confer a strong fitness against an infectious disease that produces a rapid proliferation of the advantageous mutations in a population. If indeed thrifty genes do exist, these are highly likely to be working in tandem in a polygenic manner to alter phenotype expression, where any evolutionary advantage they confer will similarly be visible only by explicitly searching for polygenic soft selection sweeps.

## Supporting Information

File S1 **File S1 includes Figure S1, Table S1 and Table S2. Figure S1.** Long haplotype forms that exhibit evidence of positive selection on chromosome 10 and chromosome 15. **Table S1.** Candidate index SNPs associated with Type 2 diabetes used in the study. **Table S2.** Candidate index SNPs associated with obesity used in the study.
(DOCX)

## References

[1] Hu FB (2011) Globalization of diabetes: the role of diet, lifestyle, and genes. Diabetes Care 34: 1249–1257. 21617109 10.2337/dc11-0442 PMC3114340

[2] Li H, Oldenburg B, Chamberlain C, O'Neil A, Xue B, et al (2012) Diabetes prevalence and determinants in adults in China mainland from 2000 to 2010: a systematic review. Diabetes Res Clin Pract 98: 226–235. 22658670 10.1016/j.diabres.2012.05.010

[3] Ma RC, Chan JC (2013) Type 2 diabetes in East Asians: similarities and differences with populations in Europe and the United States. Ann N Y Acad Sci 1281: 64–91. 23551121 10.1111/nyas.12098 PMC3708105

[4] Prentice AM, Hennig BJ, Fulford AJ (2008) Evolutionary origins of the obesity epidemic: natural selection of thrifty genes or genetic drift following predation release? Int J Obes (Lond) 32: 1607–1610. 18852700 10.1038/ijo.2008.147

[5] Speakman JR (2008) Thrifty genes for obesity, an attractive but flawed idea, and an alternative perspective: the drifty gene' hypothesis. Int J Obes (Lond) 32: 1611–1617. 18852699 10.1038/ijo.2008.161

[6] Diamond J (2003) The double puzzle of diabetes. Nature 423: 599–602. 12789325 10.1038/423599a

[7] Prentice AM, Rayco-Solon P, Moore SE (2005) Insights from the developing world: thrifty genotypes and thrifty phenotypes. Proc Nutr Soc 64: 153–161. 15960860 10.1079/pns2005421

[8] Prentice AM (2005) Early influences on human energy regulation: thrifty genotypes and thrifty phenotypes. Physiol Behav 86: 640–645. 16260008 10.1016/j.physbeh.2005.08.055

[9] Benyshek DC, Watson JT (2006) Exploring the thrifty genotype's food-shortage assumptions: a cross-cultural comparison of ethnographic accounts of food security among foraging and agricultural societies. Am J Phys Anthropol 131: 120–126. 16485298 10.1002/ajpa.20334

[10] Prentice AM (2005) Starvation in humans: evolutionary background and contemporary implications. Mech Ageing Dev 126: 976–981. 15907972 10.1016/j.mad.2005.03.018

[11] Liu X, Ong RT, Pillai EN, Elzein AM, Small KS, et al.. (2013) Detecting and Characterizing Genomic Signatures of Positive Selection in Global Populations. Am J Hum Genet. 10.1016/j.ajhg.2013.04.021 PMC3675259 23731540

[12] Altshuler DM, Gibbs RA, Peltonen L, Dermitzakis E, Schaffner SF, et al (2010) Integrating common and rare genetic variation in diverse human populations. Nature 467: 52–58. 20811451 10.1038/nature09298 PMC3173859

[13] Teo YY, Sim X, Ong RT, Tan AK, Chen J, et al (2009) Singapore Genome Variation Project: a haplotype map of three Southeast Asian populations. Genome Res 19: 2154–2162. 19700652 10.1101/gr.095000.109 PMC2775604

[14] Frazer KA, Ballinger DG, Cox DR, Hinds DA, Stuve LL, et al (2007) A second generation human haplotype map of over 3.1 million SNPs. Nature 449: 851–861. 17943122 10.1038/nature06258 PMC2689609

[15] Qian W, Deng L, Lu D, Xu S (2013) Genome-wide landscapes of human local adaptation in Asia. PLoS One 8: e54224. 23349834 10.1371/journal.pone.0054224 PMC3551950

[16] Zeggini E, Scott LJ, Saxena R, Voight BF, Marchini JL, et al (2008) Meta-analysis of genome-wide association data and large-scale replication identifies additional susceptibility loci for type 2 diabetes. Nat Genet 40: 638–645. 18372903 10.1038/ng.120 PMC2672416

[17] Ma RC, Hu C, Tam CH, Zhang R, Kwan P, et al (2013) Genome-wide association study in a Chinese population identifies a susceptibility locus for type 2 diabetes at 7q32 near PAX4. Diabetologia 56: 1291–1305. 23532257 10.1007/s00125-013-2874-4 PMC3648687

[18] Cho YS, Chen CH, Hu C, Long J, Ong RT, et al (2012) Meta-analysis of genome-wide association studies identifies eight new loci for type 2 diabetes in east Asians. Nat Genet 44: 67–72. 10.1038/ng.1019 PMC3582398 22158537

[19] Voight BF, Scott LJ, Steinthorsdottir V, Morris AP, Dina C, et al (2010) Twelve type 2 diabetes susceptibility loci identified through large-scale association analysis. Nat Genet 42: 579–589. 20581827 10.1038/ng.609 PMC3080658

[20] Perry JR, Voight BF, Yengo L, Amin N, Dupuis J, et al (2012) Stratifying type 2 diabetes cases by BMI identifies genetic risk variants in LAMA1 and enrichment for risk variants in lean compared to obese cases. PLoS Genet 8: e1002741. 22693455 10.1371/journal.pgen.1002741 PMC3364960

[21] Scott LJ, Mohlke KL, Bonnycastle LL, Willer CJ, Li Y, et al (2007) A genome-wide association study of type 2 diabetes in Finns detects multiple susceptibility variants. Science 316: 1341–1345. 17463248 10.1126/science.1142382 PMC3214617

[22] Saxena R, Voight BF, Lyssenko V, Burtt NP, de Bakker PI, et al (2007) Genome-wide association analysis identifies loci for type 2 diabetes and triglyceride levels. Science 316: 1331–1336. 17463246 10.1126/science.1142358

[23] Takeuchi F, Serizawa M, Yamamoto K, Fujisawa T, Nakashima E, et al (2009) Confirmation of multiple risk Loci and genetic impacts by a genome-wide association study of type 2 diabetes in the Japanese population. Diabetes 58: 1690–1699. 19401414 10.2337/db08-1494 PMC2699880

[24] Yamauchi T, Hara K, Maeda S, Yasuda K, Takahashi A, et al (2010) A genome-wide association study in the Japanese population identifies susceptibility loci for type 2 diabetes at UBE2E2 and C2CD4A-C2CD4B. Nat Genet 42: 864–868. 20818381 10.1038/ng.660

[25] Southam L, Soranzo N, Montgomery SB, Frayling TM, McCarthy MI, et al (2009) Is the thrifty genotype hypothesis supported by evidence based on confirmed type 2 diabetes- and obesity-susceptibility variants? Diabetologia 52: 1846–1851. 19526209 10.1007/s00125-009-1419-3 PMC2723682

[26] Wells JC (2009) Thrift: a guide to thrifty genes, thrifty phenotypes and thrifty norms. Int J Obes (Lond) 33: 1331–1338. 19752875 10.1038/ijo.2009.175

[27] Bouchard C (2007) The biological predisposition to obesity: beyond the thrifty genotype scenario. Int J Obes (Lond) 31: 1337–1339. 17356524 10.1038/sj.ijo.0803610

[28] Frayling TM (2007) Genome-wide association studies provide new insights into type 2 diabetes aetiology. Nat Rev Genet 8: 657–662. 17703236 10.1038/nrg2178

[29] Helgason A, Palsson S, Thorleifsson G, Grant SF, Emilsson V, et al (2007) Refining the impact of TCF7L2 gene variants on type 2 diabetes and adaptive evolution. Nat Genet 39: 218–225. 17206141 10.1038/ng1960

[30] Wendorf M, Goldfine ID (1991) Archaeology of NIDDM. Excavation of the “thrifty” genotype. Diabetes 40: 161–165. 10.2337/diab.40.2.161 1991567

[31] Kahn SE, Hull RL, Utzschneider KM (2006) Mechanisms linking obesity to insulin resistance and type 2 diabetes. Nature 444: 840–846. 17167471 10.1038/nature05482

[32] Watve MG, Yajnik CS (2007) Evolutionary origins of insulin resistance: a behavioral switch hypothesis. BMC Evol Biol 7: 61. 17437648 10.1186/1471-2148-7-61 PMC1868084

[33] Swinburn BA, Nyomba BL, Saad MF, Zurlo F, Raz I, et al (1991) Insulin resistance associated with lower rates of weight gain in Pima Indians. J Clin Invest 88: 168–173. 2056116 10.1172/JCI115274 PMC296017

[34] Schwartz MW, Woods SC, Porte D Jr, Seeley RJ, Baskin DG (2000) Central nervous system control of food intake. Nature 404: 661–671. 10766253 10.1038/35007534

[35] Simonis-Bik AM, Nijpels G, van Haeften TW, Houwing-Duistermaat JJ, Boomsma DI, et al (2010) Gene variants in the novel type 2 diabetes loci CDC123/CAMK1D, THADA, ADAMTS9, BCL11A, and MTNR1B affect different aspects of pancreatic beta-cell function. Diabetes 59: 293–301. 19833888 10.2337/db09-1048 PMC2797936

[36] Blyszczuk P, Czyz J, Kania G, Wagner M, Roll U, et al (2003) Expression of Pax4 in embryonic stem cells promotes differentiation of nestin-positive progenitor and insulin-producing cells. Proc Natl Acad Sci U S A 100: 998–1003. 12525695 10.1073/pnas.0237371100 PMC298715

[37] Sosa-Pineda B, Chowdhury K, Torres M, Oliver G, Gruss P (1997) The Pax4 gene is essential for differentiation of insulin-producing beta cells in the mammalian pancreas. Nature 386: 399–402. 9121556 10.1038/386399a0

