# Supplementary material for: Can Evidence from Genome-Wide Association Studies and Positive Natural Selection Surveys Be Used to Evaluate the Thrifty Gene Hypothesis in East Asians?
Source: PLoS One. 2014 Oct 22;9(10):e110974. doi: 10.1371/journal.pone.0110974 (PMC4206456; doi:10.1371/journal.pone.0110974)
Supplement: File S1 — includes Figure S1, Table S1 and Table S2. Figure S1. Long haplotype forms that exhibit evidence of positive selection on chromosome 10 and chromosome 15. Table S1. Candidate index SNPs associated with Type 2 diabetes used in the study. Table S2. Candidate index SNPs associated with obesity used in the study. (DOCX) [file pone.0110974.s001.docx]

**Supplementary**

**Figure S1.**

**A**

**chr10**

JPT

rs1111875 rs5015480


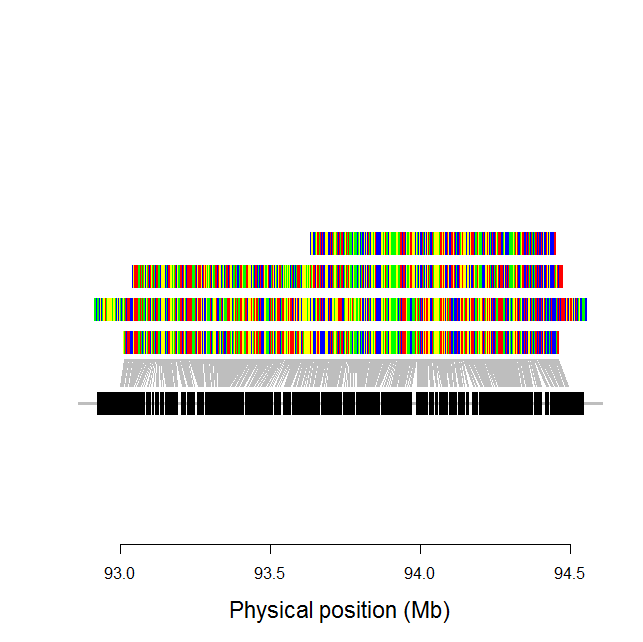


**B**

**chr15**

CHD

JPT

CHS


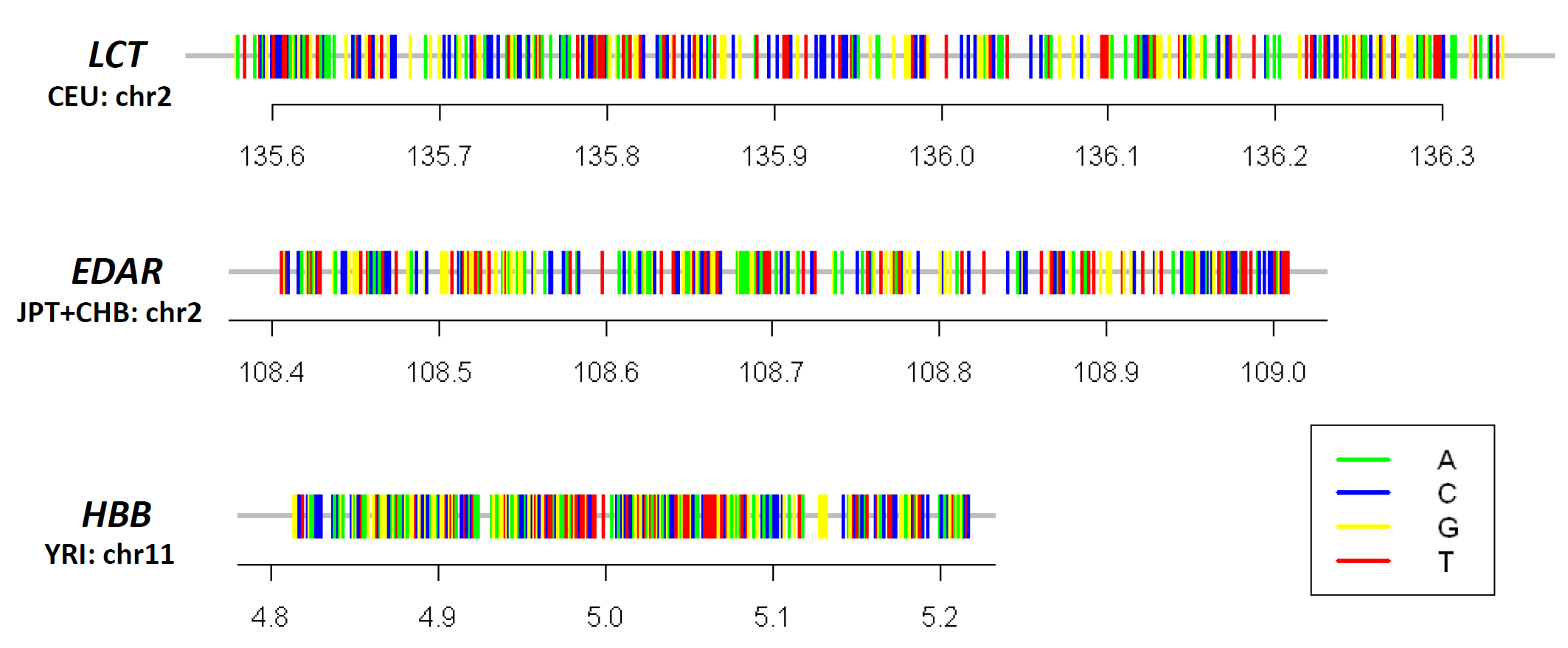

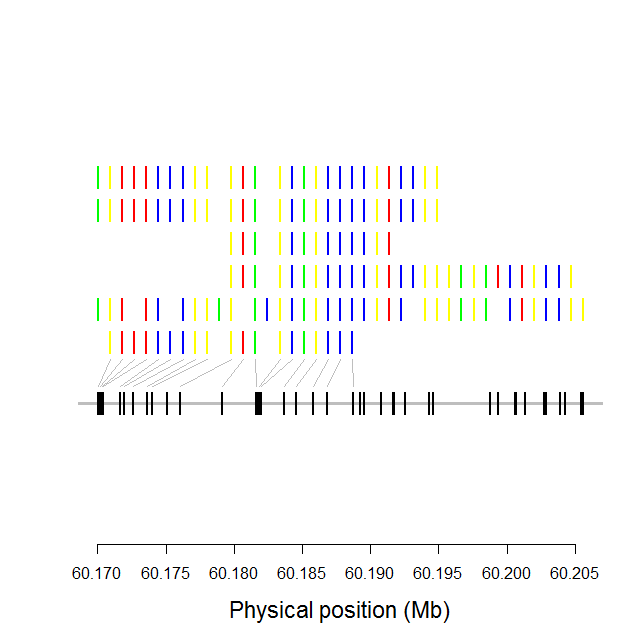


**Figure S1.** Long haplotype forms that exhibit evidence of positive selection on chromosome 10 (**panel A**) and chromosome 15 (**panel B**). The triangles indicate the positions of the trait-associated SNPs.

rs7172432

**Table S1.** Candidate index SNPs associated with Type 2 diabetes used in the study

| Chr | Genes | SNPs | Study |
| --- | --- | --- | --- |
| 1 | NOTCH2, ADAM30 | rs10923931 | [^1^](#_ENREF_1) |
| 2 | THADA | rs7578597 | [^1^](#_ENREF_1) |
| 2 | BCL11A | rs243021 | [^2^](#_ENREF_2) |
| 2 | RBM43, RND3 | rs7560163 | [^3^](#_ENREF_3) |
| 2 | RBMS1, ITGB6 | rs7593730 | [^4^](#_ENREF_4) |
| 2 | GRB14 | rs3923113 | [^5^](#_ENREF_5) |
| 2 | IRS1 | rs7578326 | [^2^](#_ENREF_2) |
| 3 | PSMD6 | rs831571 | [^6^](#_ENREF_6) |
| 3 | ADAMTS9 | rs4607103 | [^6^](#_ENREF_6) |
| 3 | IGF2BP2 | rs4402960 | [^7^](#_ENREF_7) |
|  |  | rs4402960 | [^8-10^](#_ENREF_8) |
|  |  | rs1470579 | [^11^](#_ENREF_11)^,^[^2^](#_ENREF_2) |
|  |  | rs6769511 | [^12^](#_ENREF_12) |
| 3 | ST6GAL1 | rs16861329 | [^5^](#_ENREF_5) |
| 4 | MGC21675 | rs7656416 | [^13^](#_ENREF_13) |
| 4 | MAEA | rs6815464 | [^6^](#_ENREF_6) |
| 4 | WFS1 | rs1801214 | [^2^](#_ENREF_2) |
| 5 | ZBED3 | rs4457053 | [^2^](#_ENREF_2) |
| 6 | CDKAL1 | rs4712523 | [^14^](#_ENREF_14) |
|  |  | rs4712524 | [^12^](#_ENREF_12) |
|  |  | rs10946398 | [^10^](#_ENREF_10) |
|  |  | rs7754840 | [^8^](#_ENREF_8)^,^[^9^](#_ENREF_9)^,^[^15^](#_ENREF_15) |
|  |  | rs7756992 | [^16^](#_ENREF_16) |
|  |  | rs7766070 | [^7^](#_ENREF_7) |
|  |  | rs10440833 | [^2^](#_ENREF_2) |
|  |  | rs6931514 | [^1^](#_ENREF_1) |
| 6 | ZFAND3 | rs9470794 | [^6^](#_ENREF_6) |
| 6 | KCNK16 | rs1535500 | [^6^](#_ENREF_6) |
| 6 | C6orf57 | rs1048886 | [^17^](#_ENREF_17) |
| 7 | JAZF1 | rs864745 | [^1^](#_ENREF_1) |
|  |  | rs849134 | [^2^](#_ENREF_2) |
| 7 | PAX4 | rs6467136 | [^6^](#_ENREF_6) |
|  |  | rs10229583 | [^18^](#_ENREF_18) |
| 7 | KLF14 | rs972283 | [^6^](#_ENREF_6) |
| 8 | ANK1 | rs515071 | [^13^](#_ENREF_13) |
| 8 | TP53INP1 | rs896854 | [^6^](#_ENREF_6) |
| 8 | SLC30A8 | rs13266634 | [^8-10^](#_ENREF_8)^,^[^14^](#_ENREF_14) |
|  |  | rs3802177 | [^2^](#_ENREF_2)^,^[^7^](#_ENREF_7) |
| 9 | GLIS3 | rs7041847,rs10814916 | [^6^](#_ENREF_6) |
|  |  | rs10814916 | [^15^](#_ENREF_15) |
| 9 | PTPRD | rs17584499 | [^19^](#_ENREF_19) |
| 9 | CDKN2A, CDKN2B | rs2383208 | [^14^](#_ENREF_14)^,^[^15^](#_ENREF_15) |
|  |  | rs10965250 | [^2^](#_ENREF_2) |
|  |  | rs10811661 | [^8^](#_ENREF_8)^,^[^9^](#_ENREF_9) |
|  |  | rs1333051 | [^20^](#_ENREF_20) |
|  |  | rs7018475 | [^21^](#_ENREF_21) |
| 9 | CHCHD9 | rs13292136 | [^2^](#_ENREF_2) |
| 10 | CDC123 | rs11257655 | [^15^](#_ENREF_15) |
|  |  | rs10906115 | [^22^](#_ENREF_22) |
|  |  | rs12779790 | [^1^](#_ENREF_1) |
| 10 | VPS26A | rs1802295 | [^5^](#_ENREF_5) |
| 10 | HHEX | rs1111875 | [^8^](#_ENREF_8)^,^[^9^](#_ENREF_9)^,^[^14^](#_ENREF_14) |
|  |  | rs5015480 | [^2^](#_ENREF_2)^,^[^7^](#_ENREF_7) |
| 10 | TCF7L2 | rs7901695 | [^10^](#_ENREF_10) |
|  |  | rs4506565 | [^23^](#_ENREF_23) |
|  |  | rs7903146 | [^1^](#_ENREF_1)^,^[^2^](#_ENREF_2)^,^[^7-9^](#_ENREF_7)^,^[^11^](#_ENREF_11)^,^[^14^](#_ENREF_14)^,^[^16^](#_ENREF_16)^,^[^24-28^](#_ENREF_24) |
| 10 | GRK5 | rs10886471 | [^15^](#_ENREF_15) |
| 11 | KCNQ1 | rs231362 | [^2^](#_ENREF_2) |
|  |  | rs2237892 | [^14^](#_ENREF_14)^,^[^29^](#_ENREF_29) |
|  |  | rs163182 | [^19^](#_ENREF_19)^,^[^30^](#_ENREF_30) |
|  |  | rs2237895 | [^19^](#_ENREF_19) |
|  |  | rs2237897 | [^12^](#_ENREF_12) |
|  |  | rs5215 | [^10^](#_ENREF_10) |
|  |  | rs5219 | [^9^](#_ENREF_9)^,^[^26^](#_ENREF_26) |
| 11 | CENTD2 | rs1552224 | [^2^](#_ENREF_2) |
| 11 | MTNR1B | rs1387153 | [^2^](#_ENREF_2) |
| 12 | HMGA2 | rs1531343 | [^2^](#_ENREF_2) |
| 12 | TSPAN8,LGR5 | rs7961581 | [^1^](#_ENREF_1) |
| 12 | HNF1A | rs7305618,rs7957197 | [^20^](#_ENREF_20) |
|  |  | rs7957197 | [^2^](#_ENREF_2) |
| 13 | SGCG, SACS | rs9552911 | [^11^](#_ENREF_11) |
| 13 | SPRY2 | rs1359790 | [^22^](#_ENREF_22) |
| 15 | RASGRP1 | rs7403531 | [^15^](#_ENREF_15) |
| 15 | C2CD4A,C2CD4B | rs7172432 | [^31^](#_ENREF_31) |
| 15 | HMG20A | rs7178572 | [^5^](#_ENREF_5)^,^[^7^](#_ENREF_7) |
|  |  | rs7178572 | [^5^](#_ENREF_5) |
| 15 | ZFAND6 | rs11634397 | [^2^](#_ENREF_2) |
| 15 | AP3S2 | rs2028299 | [^5^](#_ENREF_5) |
| 15 | PRC1 | rs8042680 | [^2^](#_ENREF_2) |
| 16 | FTO | rs8050136 | [^9^](#_ENREF_9)^,^[^10^](#_ENREF_10)^,^[^26^](#_ENREF_26) |
|  |  | rs9939609 | [^7^](#_ENREF_7) |
|  |  | rs11642841 | [^2^](#_ENREF_2) |
| 17 | SRR | rs391300 | [^19^](#_ENREF_19) |
| 17 | HNF1B | rs4430796 | [^15^](#_ENREF_15) |
| 18 | LAMA1 | rs8090011 | [^7^](#_ENREF_7) |
| 19 | PEPD | rs3786897 | [^6^](#_ENREF_6) |
| 20 | FITM2,R3HDML,HNF4A | rs6017317 | [^6^](#_ENREF_6) |
| 20 | HNF4A | rs4812829 | [^5^](#_ENREF_5) |
| 23 | FAM58A | rs12010175 | [^15^](#_ENREF_15) |
| 23 | DUSP9 | rs5945326 | [^2^](#_ENREF_2)^,^[^15^](#_ENREF_15) |

**Table S2.** Candidate index SNPs associated with obesity used in the study

| Chr | Genes | SNPs | Study |
| --- | --- | --- | --- |
| 10 | KCNMA1 | rs2116830 | [^32^](#_ENREF_32) |
| 13 | OLFM4 | rs9568856 | [^33^](#_ENREF_33) |
| 14 | NRXN3 | rs11624704 | [^34^](#_ENREF_34) |
| 16 | FTO | rs17817449 | [^34^](#_ENREF_34) |
| 17 | HOXB5 | rs9299 | [^33^](#_ENREF_33) |
| 18 | MC4R | rs17782313 | [^35^](#_ENREF_35) |
| 21 | NCAM2 | rs11088859 | [^34^](#_ENREF_34) |

**References**

1. Zeggini, E. *et al.* Meta-analysis of genome-wide association data and large-scale replication identifies additional susceptibility loci for type 2 diabetes. *Nat Genet* **40**, 638-45 (2008).

2. Voight, B.F. *et al.* Twelve type 2 diabetes susceptibility loci identified through large-scale association analysis. *Nat Genet* **42**, 579-89 (2010).

3. Palmer, N.D. *et al.* A genome-wide association search for type 2 diabetes genes in African Americans. *PLoS One* **7**, e29202 (2012).

4. Qi, L. *et al.* Genetic variants at 2q24 are associated with susceptibility to type 2 diabetes. *Hum Mol Genet* **19**, 2706-15 (2010).

5. Kooner, J.S. *et al.* Genome-wide association study in individuals of South Asian ancestry identifies six new type 2 diabetes susceptibility loci. *Nat Genet* **43**, 984-9 (2011).

6. Cho, Y.S. *et al.* Meta-analysis of genome-wide association studies identifies eight new loci for type 2 diabetes in east Asians. *Nat Genet* **44**, 67-72 (2011).

7. Perry, J.R. *et al.* Stratifying type 2 diabetes cases by BMI identifies genetic risk variants in LAMA1 and enrichment for risk variants in lean compared to obese cases. *PLoS Genet* **8**, e1002741 (2012).

8. Saxena, R. *et al.* Genome-wide association analysis identifies loci for type 2 diabetes and triglyceride levels. *Science* **316**, 1331-6 (2007).

9. Scott, L.J. *et al.* A genome-wide association study of type 2 diabetes in Finns detects multiple susceptibility variants. *Science* **316**, 1341-5 (2007).

10. Zeggini, E. *et al.* Replication of genome-wide association signals in UK samples reveals risk loci for type 2 diabetes. *Science* **316**, 1336-41 (2007).

11. Saxena, R. *et al.* Genome-wide association study identifies a novel locus contributing to type 2 diabetes susceptibility in Sikhs of Punjabi origin from India. *Diabetes* **62**, 1746-55 (2013).

12. Unoki, H. *et al.* SNPs in KCNQ1 are associated with susceptibility to type 2 diabetes in East Asian and European populations. *Nat Genet* **40**, 1098-102 (2008).

13. Imamura, M. *et al.* A single-nucleotide polymorphism in ANK1 is associated with susceptibility to type 2 diabetes in Japanese populations. *Hum Mol Genet* **21**, 3042-9 (2012).

14. Takeuchi, F. *et al.* Confirmation of multiple risk Loci and genetic impacts by a genome-wide association study of type 2 diabetes in the Japanese population. *Diabetes* **58**, 1690-9 (2009).

15. Li, H. *et al.* A genome-wide association study identifies GRK5 and RASGRP1 as type 2 diabetes loci in Chinese Hans. *Diabetes* **62**, 291-8 (2013).

16. Steinthorsdottir, V. *et al.* A variant in CDKAL1 influences insulin response and risk of type 2 diabetes. *Nat Genet* **39**, 770-5 (2007).

17. Sim, X. *et al.* Transferability of type 2 diabetes implicated Loci in multi-ethnic cohorts from southeast Asia. *PLoS Genet* **7**, e1001363 (2011).

18. Ma, R.C. *et al.* Genome-wide association study in a Chinese population identifies a susceptibility locus for type 2 diabetes at 7q32 near PAX4. *Diabetologia* **56**, 1291-305 (2013).

19. Tsai, F.J. *et al.* A genome-wide association study identifies susceptibility variants for type 2 diabetes in Han Chinese. *PLoS Genet* **6**, e1000847 (2010).

20. Parra, E.J. *et al.* Genome-wide association study of type 2 diabetes in a sample from Mexico City and a meta-analysis of a Mexican-American sample from Starr County, Texas. *Diabetologia* **54**, 2038-46 (2011).

21. Huang, J., Ellinghaus, D., Franke, A., Howie, B. & Li, Y. 1000 Genomes-based imputation identifies novel and refined associations for the Wellcome Trust Case Control Consortium phase 1 Data. *Eur J Hum Genet* **20**, 801-5 (2012).

22. Shu, X.O. *et al.* Identification of new genetic risk variants for type 2 diabetes. *PLoS Genet* **6**, e1001127 (2010).

23. Genome-wide association study of 14,000 cases of seven common diseases and 3,000 shared controls. *Nature* **447**, 661-78 (2007).

24. Tabassum, R. *et al.* Genome-wide association study for type 2 diabetes in Indians identifies a new susceptibility locus at 2q21. *Diabetes* **62**, 977-86 (2013).

25. Kho, A.N. *et al.* Use of diverse electronic medical record systems to identify genetic risk for type 2 diabetes within a genome-wide association study. *J Am Med Inform Assoc* **19**, 212-8 (2012).

26. Timpson, N.J. *et al.* Adiposity-related heterogeneity in patterns of type 2 diabetes susceptibility observed in genome-wide association data. *Diabetes* **58**, 505-10 (2009).

27. Salonen, J.T. *et al.* Type 2 diabetes whole-genome association study in four populations: the DiaGen consortium. *Am J Hum Genet* **81**, 338-45 (2007).

28. Sladek, R. *et al.* A genome-wide association study identifies novel risk loci for type 2 diabetes. *Nature* **445**, 881-5 (2007).

29. Yasuda, K. *et al.* Variants in KCNQ1 are associated with susceptibility to type 2 diabetes mellitus. *Nat Genet* **40**, 1092-7 (2008).

30. Cui, B. *et al.* A genome-wide association study confirms previously reported loci for type 2 diabetes in Han Chinese. *PLoS One* **6**, e22353 (2011).

31. Yamauchi, T. *et al.* A genome-wide association study in the Japanese population identifies susceptibility loci for type 2 diabetes at UBE2E2 and C2CD4A-C2CD4B. *Nat Genet* **42**, 864-8 (2010).

32. Jiao, H. *et al.* Genome wide association study identifies KCNMA1 contributing to human obesity. *BMC Med Genomics* **4**, 51 (2011).

33. Bradfield, J.P. *et al.* A genome-wide association meta-analysis identifies new childhood obesity loci. *Nat Genet* **44**, 526-31 (2012).

34. Wang, K. *et al.* A genome-wide association study on obesity and obesity-related traits. *PLoS One* **6**, e18939 (2011).

35. Meyre, D. *et al.* Genome-wide association study for early-onset and morbid adult obesity identifies three new risk loci in European populations. *Nat Genet* **41**, 157-9 (2009).
